# Direct valorization of agricultural byproducts to vanillin by a synergistic *Komagataella phaffii* co-culture system

**DOI:** 10.1186/s13068-026-02770-6

**Published:** 2026-05-19

**Authors:** Difei Zhou, Yuji Yang, Yi Shao, Lina Sun, Geqi Qi, Menghui Tao, Zhen Li, Fengjiao Xin

**Affiliations:** 1 Laboratory of Biomanufacturing and Food Engineering, Institute of Food Science and Technology, Chinese Academy of Agricultural Sciences, Beijing, 100193 China; 2 Institute of Food Science Technology Nutrition and Health (Cangzhou), Chinese Academy of Agricultural Sciences, Cangzhou, 061001 China; 3 Institute of Agricultural and Animal Husbandry of Hinggan League, Inner Mongolia Innovation Center of Biological Breeding Technology, Inner Mongolia Key Laboratory of Rice Breeding Innovation in Northern Cold Regions, Ulanhot, 137400 Inner Mongolia China

**Keywords:** Agricultural byproducts, Co-culture, *Komagataella phaffii*, Xylan hydrolysis, Vanillin biosynthesis

## Abstract

**Background:**

The annual abundance of lignocellulosic agricultural byproducts poses a significant environmental burden. Leveraging this underutilized biomass to produce high-value, food-grade products represents a promising strategy for diversifying food ingredient sources and advancing the principles of the circular bioeconomy.

**Results:**

Here, a novel *Komagataella phaffii* co-culture system was developed for integrated xylan hydrolysis and vanillin biosynthesis. In the upstream xylan hydrolysis module, overexpression of a novel multi-modular bifunctional xylanase/feruloyl esterase enabled the direct hydrolysis of xylans in agricultural byproducts to release ferulic acid (FA), thereby providing precursors for the downstream vanillin synthesis module. In the downstream module, FA was converted to vanillin via heterologous expression of feruloyl-CoA synthetase (FCS) and enoyl-CoA hydratase/aldolase (ECH). Subsequent optimization of strain inoculation ratios and carbon source composition in the co-culture system increased the titer of vanillin and its derivatives to 2.63 mM, achieving a 47.90% molar conversion rate from ultrafine-grinding corn cob (UGCC).

**Conclusions:**

This study establishes a scalable platform for synthesizing aromatic compounds from low-cost lignocellulosic feedstocks, offering a strategic blueprint for valorizing agricultural residues.

## Introduction

Lignocellulose, the primary component of plant-based agricultural byproducts, is the most abundant renewable biomass on Earth, with global annual production estimated at approximately 180 billion tons [1, 2]. Despite its abundance, a substantial proportion of lignocellulosic biomass is currently disposed of through incineration or uncontrolled discarding, leading to resource waste and environmental degradation [3]. As the dominant hemicellulose constituent in lignocellulose, xylan plays a crucial role in maintaining the structural stability of plant cell walls [4–6]. Ferulic acid (FA) is one of the most abundant hydroxycinnamic acids in plant cell walls, where it is predominantly ester-linked to arabinoxylan side chains [7]. The content of FA in common agricultural residues, such as corn cobs, wheat bran, and rice straw, typically ranges from 3 to 15 mg/g dry biomass, depending on the plant species and tissue [8]. As a natural precursor of vanillin, FA can be released from lignocellulosic biomass by the synergistic action of xylanase and feruloyl esterase, offering a renewable and sustainable route for vanillin production [9]. This heteropolymer, rich in carbohydrates and aromatic moieties, is recognized as an ideal renewable feedstock for producing biofuels, high-value chemicals, and functional materials [10]. Therefore, the bioconversion of xylan into value-added products has emerged as a promising strategy for lignocellulosic biorefinery, accelerating the transition toward a circular bioeconomy.

With the rapid advancement of synthetic biology, microbial chassis strains have been engineered as efficient cell factories for the production of high-value natural products. By integrating heterologous pathways, rewiring native metabolism, and optimizing enzyme expression, these microbial platforms enable the efficient conversion of low-cost renewable substrates into aromatic compounds and other specialty chemicals [11, 12]. Notably, recent advances have enabled the direct conversion of xylan into aromatic products using engineered microbes. A recent study conducted by Zhu et al. developed a novel cell factory for ρ-coumaric acid (ρ-CA) production in *Yarrowia lipolytica*, achieving a yield of 65.30 mg/L ρ-CA using bagasse-derived xylan as the carbon source [13]. However, current strategies that rely on maximizing pathway integration for one-pot bioconversion face critical bottlenecks. Excessive metabolic burden, imbalanced expression of key enzymes, and low conversion efficiency collectively limit the performance of engineered cell factories [14]. A rationally designed co-culture system based on modular engineering may hold the key to addressing the aforementioned bottlenecks [15]. The co-culture system establishes an optimal cellular microenvironment that alleviates metabolic stress burden on the chassis cells, balances biosynthetic flux, minimizes pathway crosstalk, and facilitates metabolite cross-feeding, thereby synergistically enhancing pathway efficiency and metabolic robustness, and ultimately maximizing the production rate and yield of the entire biomanufacturing system [16, 17]. Modular co-culture systems have been successfully implemented for high-value aromatic compounds biosynthesis. For instance, Jones et al. engineered an *Escherichia coli* co-culture system by partitioning the flavonoid biosynthesis pathway into an upstream malonyl-CoA-dependent module and a downstream NADPH-dependent module, achieving a 970-fold flavonoid yield enhancement over monocultures through subsequent fermentation optimization [18]. Wang et al. developed a cross-kingdom consortium, *E. coli–Candida glycerinogenes*, for sustainable caffeic acid (CA) production from sugarcane bagasse hydrolysate, achieving a CA titer of 1943.20 mg/L [19]. Besides, Chacόn M et al. designed a synthetic co-culture platform comprising an engineered *Bacillus subtilis* hydroxycinnamic acid inducible hydrolysis module and an engineered *E. coli* biotransformation module, which directly converts lignocellulose-incorporated FA and coumaric acid into coniferol and chavicol, demonstrating efficient valorization of agricultural residues [20].

Vanillin, a globally dominant aromatic flavorant, is extensively used in food, pharmaceuticals, and cosmetic industries, with annual demand exceeding 20,000 tons [21, 22]. Natural vanillin extraction from vanilla orchids remains constrained by low yields and high costs [23, 24], while chemical synthesis, accounting for 90% of global supply, faces challenges including high energy consumption, environmental pollution, and excessive byproduct generation [25]. In contrast, vanillin biosynthesis offers a sustainable alternative based on renewable feedstock, short production cycles, and eco-friendly processes [26]. Theoretically, under the synergistic action of xylanase and feruloyl esterase (FAE), xylan can be enzymatically hydrolyzed into xylose and xylo-oligosaccharides (XOS), with simultaneous release of FA [27]. Catalyzed by feruloyl-CoA synthetase (FCS) and enoyl-CoA hydratase (ECH), FA can be subsequently converted to vanillin in the CoA-dependent bioconversion pathway [28]. However, there have been no reports on the development of a co-culture system for the efficient conversion of agricultural byproducts into vanillin.

**Fig. 1:**
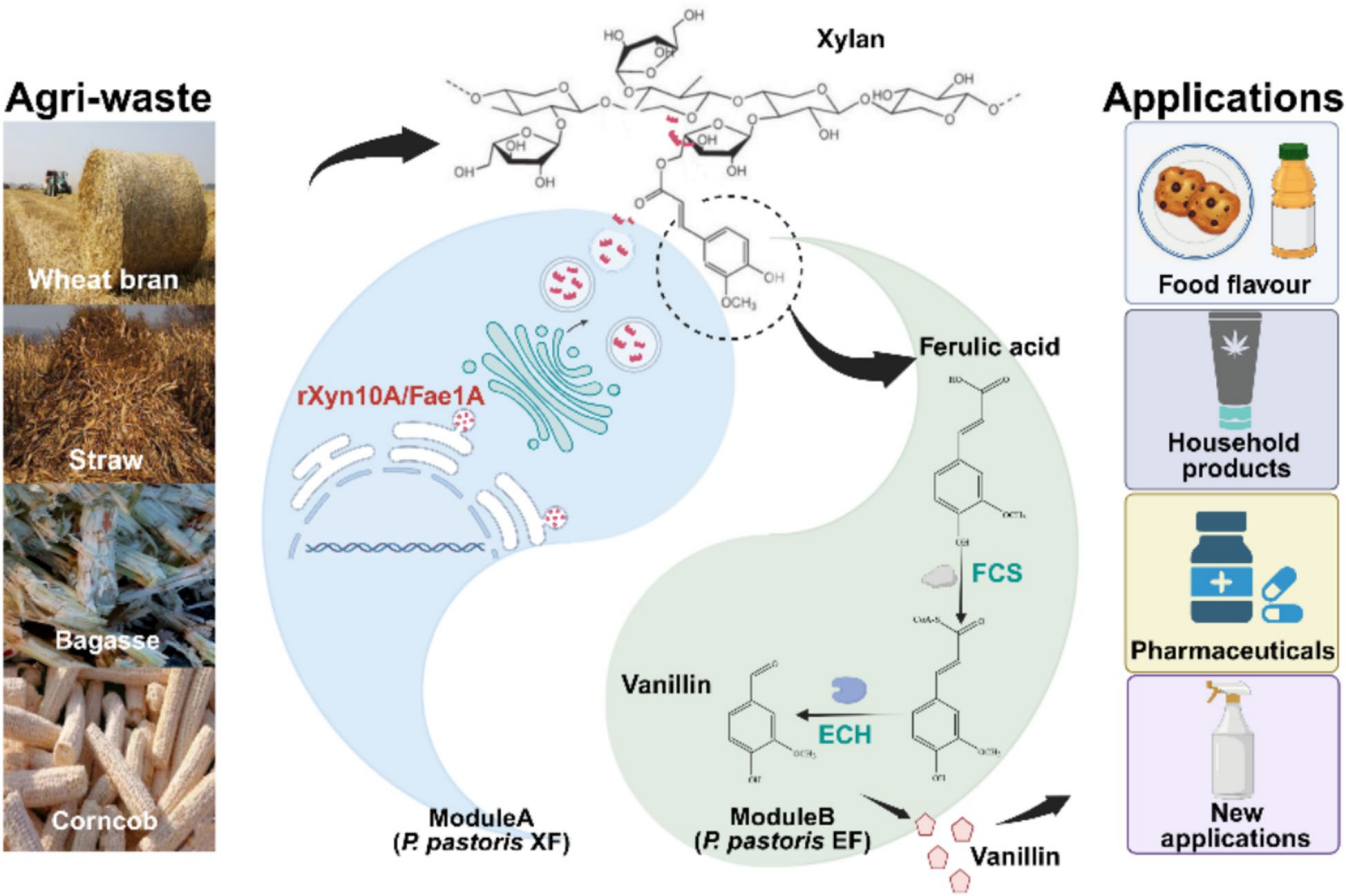
Schematic representation of a modular co-culture system for the direct conversion of lignocellulosic biomass (UGCC) into vanillin (VA). The upstream module employs a recombinant strain expressing the bifunctional enzyme rXyn10A/Fae1A to degrade hemicellulose and release ester-bound ferulic acid (FA). The downstream module converts FA into VA via an engineered biosynthetic pathway. By spatially separating biomass depolymerization and product biosynthesis, this co-culture system reduces metabolic burden and improves pathway efficiency, enabling a one-pot bioconversion of raw biomass into value-added aromatics

In this work, a novel *K. phaffii* (formerly *Pichia pastoris*) co-culture system was developed for integrated xylan hydrolysis and vanillin biosynthesis, aiming to enhance aromatic compound production from agricultural byproducts through advanced engineering strategies (Fig. 1). *K. phaffii*, a Generally Recognized As Safe (GRAS)-certified strain characterized by robust genetic stability, a well-defined genetic profile, strong post-translational modification capacity, and high-density fermentation suitability, was employed as the chassis strain to enable efficient vanillin bioproduction [29]. Specifically, two functional modules were constructed: an upstream xylan hydrolysis module for depolymerizing lignocellulosic substrates and a downstream vanillin production module for converting FA to vanillin. Key fermentation parameters, including initial inter-module inoculation ratio and carbon source composition, were systematically optimized to maximize pathway synergy. This integrated platform demonstrates efficient valorization of agricultural byproducts into high-value vanillin, providing a sustainable alternative to conventional production methods.

## Materials and methods

### Plasmids and strain construction

The microbial strains, plasmids, and primers used in this study are listed in Tables 1 and 2. The gene encoding the bifunctional enzyme rXyn10A/Fae1A, as well as the *ech* and *fcs* genes, were optimized based on the codon preference in *K. phaffii* and were synthesized by Beijing Genomics Institute (BGI, China). The vectors pPICZαA and pPIC3.5 K were purchased from Thermo (USA). The *rXyn10A/Fae1A* gene was cloned into the secretion vector pPICZαA. The recombinant plasmid pPICZαA-rXyn10A/Fae1A was linearized with the *Sac I* restriction enzyme and then transformed into *K. phaffii* GS115 using the Gene Pulser Xcell Electroporation System (BIO-RAD, USA). The transformants were selected on a Yeast-extract Peptone Dextrose (YPD) agar plate containing 300 μg/mL of Zeocin, and the positive colonies were screened through colony PCR with primers YAN-*rXyn10A/Fae1A-*F and YAN-*rXyn10A/Fae1A-*R. The *ech* and *fcs* genes were cloned into the vector pPIC3.5 K. The recombinant plasmid pPIC3.5 K-*ech*-*fcs* was linearized with the *BspE I* restriction enzyme and then transformed into *K. phaffii* GS115 by electroporation. The transformants were selected on MD plates without histidine and verified by colony PCR with primers YAN-*ech-*F/R, and YAN-*fcs-*F/R. *E. coli* Top 10 was used as the cloning host for plasmid construction.

**Table 1:** Strains and plasmids used in this study

Strain/plasmid	Relevant characteristics	Source
Strains		
*K. phaffii* GS115	Mut + , his4^−^, AOX1, AOX2	Invitrogen
XF	GS115:: AOX1:: P_AOX1_-*rXyn10A/Fae1A*-T_AOX1_	This study
EF	GS115:: HIS4:: P_AOX1_-*ech*-*fcs*-T_AOX1_	This study
Plasmids		
pPICZαA	Zeor, *K. phaffii* expression vector	Invitrogen
pPIC3.5 K	G418, *K. phaffii* expression vector	Invitrogen
pPICZαA*-rXyn10A/Fae1A*	Zeor, the plasmid carrying *rXyn10A/Fae1A* gene	This study
pPIC3.5 K-*ech*-*fcs*	G418, the plasmid carrying *ech-fcs* gene	This study

**Table 2:** Primers used in this study

Primers	Sequences (5ʹ-3ʹ)
YAN-*rXyn10A/Fae1A-*F	ATGAGGGCTTTCTGAGTG
YAN-*rXyn10A/Fae1A-*R	GTTATCGTAGGCGTTCTT
YAN-*ech-*F	AAGTAATCCTCGGCTTGTT
YAN-*ech-*R	TTACTTCTCTGGGTCGAAAG
YAN-*fcs-*F	TCCACAGGTCCATTCTCA
YAN-*fcs-*R	TTCTCGTAAGTGCCCAAC

### Culture conditions

Single clones of *K. phaffii* strains were inoculated into YPD medium (10 g/L yeast extract, 20 g/L peptone and 20 g/L glucose) and cultivated overnight at 30℃ with shaking at 220 rpm until the OD_600_ reached 2–6. Then, the overnight-cultured seed was inoculated into buffered glycerol-complex medium (BMGY) containing 10 g/L yeast extract, 20 g/L peptone, 3 g/L K_2_HPO_4_, 11.8 g/L KH_2_PO_4_, 100 mL of 10 × yeast nitrogen base (YNB), 2 mL of 500 × biotin (4 × 10^–4^ g/L), and 10 mL of glycerol. Cultivation continued under the same conditions until the OD_600_ reached 4–5. The cultures were then left to stand overnight at 4℃, and the precipitated cells were resuspended in BMMY medium (adjusted to OD_600_ = 3–4) for continued cultivation at 30℃ with shaking at 220 rpm. The formula of buffered methanol-complex medium (BMMY) consists of 10 g/L yeast extract, 20 g/L peptone, 3 g/L K_2_HPO_4_, 11.8 g/L KH_2_PO_4_, 100 mL of 10 × YNB, 2 mL of 500 × biotin, and 5 mL of methanol. Methanol was added every 24 h to a final concentration of 1.5% to induce the expression of the exogenous genes. For co-culture, after each strain was grown in BMGY medium, they were resuspended and adjusted to the same OD_600_ value using a mixture of BMMY medium in a volume ratio of 1:1. Low-Luria–Bertani (LLB) medium containing 10 g/L tryptone, 5 g/L sodium chloride, and 5 g/L yeast extract was used for the plasmid amplification. YPD agar plates supplemented with the corresponding antibiotic resistance were used for screening recombinant strains.

### Pretreatment of agricultural residues

The agricultural byproducts utilized in this study originated from the local market in Beijing, China. De-starched wheat bran (DSWB) was prepared according to the method of Xu et al. [30] and subsequently ground and passed through a 50-mesh sieve, corresponding to particle sizes of less than 300 μm. Steam-exploded corn cob (SECC) and steam-exploded corn stalk (SECS) were prepared following the procedure reported by Liu et al. [31] and consisted of irregular fibrous fragments with sizes typically in the sub-millimeter to millimeter range. Ultrafine-grinding corn cob (UGCC), ultrafine-grinding corn stalk (UGCS), ultrafine-grinding rice straw (UGRS), and ultrafine-grinding wheat straw (UGWS) were prepared using the method established by Li et al. [32], resulting in a median particle size of approximately 19.65 μm. FA in the pretreated agricultural byproducts was quantified using the alkaline hydrolysis method. Specifically, 0.005 g of the substrate was weighed and mixed with 1 mL of 2 M NaOH and incubated with agitation at room temperature for 5 h. Afterwards, the pH was adjusted to a range of 6–7 using HCl, and the final volume was adjusted to 2 mL with deionized water. Subsequently, the mixture was centrifuged at 12,000 ×*g* for 10 min to collect the supernatant, which was then combined with an equal volume of ethanol, and the resulting mixture was used to detect the concentration of FA using high-performance liquid chromatography (HPLC). The results of alkaline hydrolysis served as a reference for the theoretical maximum FA release from each substrate.

### Fermentation product analysis

Cell growth was monitored by measuring optical density at 600 nm (OD₆₀₀) using a UVmini-1280 spectrophotometer (Shimadzu, Japan). After fermentation, the broths were centrifuged at 12,000 ×*g* for 30 min at 4 °C to separate cells from the supernatant. For analysis, 500 μL of cell-free culture supernatant was mixed with an equal volume of absolute ethanol and filtered through a 0.22-μm syringe filter. The concentrations of FA, vanillin and its derivatives were quantified using an Agilent 1220 HPLC system equipped with a variable wavelength detector (VWD, Agilent) and a ZORBAX SB-C18 column (5 µm, 4.6 × 250 mm, Agilent) at 280 nm at room temperature. The mobile phase consisted of A (ddH_2_O containing 0.1% formic acid) and B (methanol containing 0.1% formic acid). The injection volume was 10 μL and the flow rate was 0.5 mL/min. The gradient elution conditions were as follows: 0–8.5 min, 60% A in B; 9.5–18 min, 15% A in B, 19–25 min, 60% A in B.

Reducing sugars were analyzed by thin layer chromatography (TLC) on a silica gel TLC, using a mobile phase of n-butanol, ethanol, and water (5:3:2, v/v/v). The developer solution consisted of sulfuric acid and methanol in a ratio of 5:95 (v/v). For quantitative analysis, reducing sugar content was determined using the 3,5-dinitrosalicylic acid (DNS) method, measuring the absorbance at 540 nm with a UVmini-1280 spectrophotometer (Shimadzu, Japan). Xylose and XOS were further analyzed by high-performance anion exchange chromatography with pulsed amperometric detection (HPAEC-PAD) on a DIONX ICS-3000 system (Thermo Scientific, Waltham, USA), equipped with a CarboPac™ PA10 analytical column (4 × 250 mm). The mobile phase consisted of A (ddH_2_O) and B (200 mM NaOH) at a flow rate of 1 mL/min. The gradient elution conditions were as follows: 0–30 min, 91% A in B; 30–70 min, 45% A in B; 70–70.1 min, 20% A in B; 70.1–80 min, 20% A in B, 80–80.1 min, 91% A in B; 80.1–90 min, 91% A in B.

### Scanning electron microscopy (SEM) analysis

Surface morphology was analyzed using an SEM (JSM-7401, HITACHI, Japan). The samples were gently rinsed with phosphate-buffered solution (PBS) three times and collected by centrifugation at 3000 ×*g* for 10 min at 4 ℃. The pellets were then fixed with a 3.5% glutaraldehyde solution at room temperature in the dark for 48 h. The fixed samples were rinsed three times with PBS, with each rinse lasting more than 5 min, then dehydrated in ethanol solution (30%, 50%, 60%, 70%, 80%, 90%, 95%, and 100%), with each step performed for 5 min. After air-drying, the samples were sputter-coated with a 10 nm layer of gold using a magnetron sputter coater (Model EIKO IB-5, Eiko Inc., Japan). Finally, the surface morphology of the samples was observed using SEM at an accelerating voltage of 10 kV.

### Fourier transform infrared spectroscopy (FTIR) analysis

An FTIR spectrometer (VERTEX 70, Bruker, Germany) was employed to examine the structural modifications in the lignocellulosic agricultural byproducts resulting from fermentation treatments. The samples treated with different fermentation durations were oven-dried to remove moisture, and finely ground in a mortar and thoroughly mixed with KBr in a weight ratio of 1:100. The mixture was then pressed into pellets for FTIR analysis. The spectra were recorded in transmittance mode, ranging from 4000 cm⁻^1^ to 400 cm⁻^1^, with a spectral resolution of 4 cm⁻^1^. Each sample was scanned 64 times, and all measurements were performed in triplicate to ensure the comparability of the FTIR spectra.

### Statistical significance

Statistical significance between the groups was determined through one-way analysis of variance (ANOVA) followed by Duncan’s multiple range test (*p* < 0.05). For statistical analysis, the results of three independent experiments were compared. Statistical analyses were performed using SPSS, version 26. All experiments were performed in triplicate. The values are presented as the mean and standard deviation.

## Results and discussion

### Construction of the upstream xylan-hydrolysis module

**Fig. 2:**
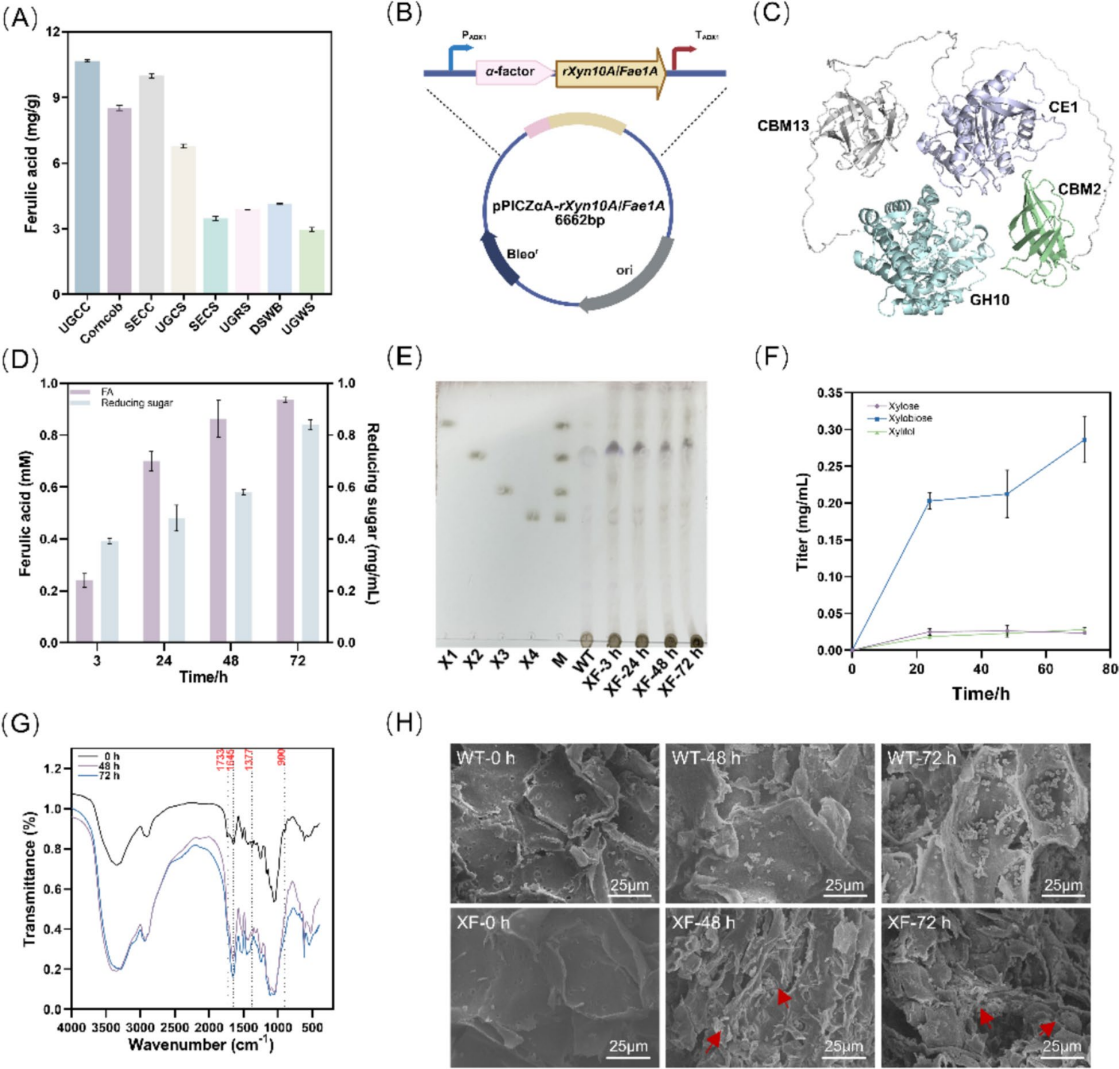
Construction of the upstream xylan-hydrolysis module strain XF and xylan hydrolysis capacity and product analysis of strain XF. **A** Determination of FA content in pretreated natural substrates using alkaline hydrolysis method. **B** Schematic diagram of recombinant plasmid pPICZαA-*rXyn10A/Fae1A*. **C** Ab initio structure prediction of rXyn10A/Fae1A using AlphaFold3. **D** Concentration of FA and reducing sugars. **E** Qualitative analysis of reducing sugars by TLC. **F** Quantitative analysis of reducing sugars by HPAEC-PAD. **G** FTIR spectral analysis. **H** SEM characterization of substrate surface morphology

The release of FA from agricultural byproducts represents a critical initial step in valorizing lignocellulosic biomass into high-value vanillin. This process depends on both effective substrate pretreatment and efficient enzymatic hydrolysis. In this study, steam explosion and ultrafine grinding were employed as pretreatment methods to reduce the crystalline structure of lignocellulose, increase the substrate’s specific surface area, and enhance degradation efficiency [31, 32]. The ultrafine-grinding corncob (UGCC) exhibited the highest FA release amount among the tested agricultural byproducts after pretreatment, including corn cobs, corn stover, rice straw, wheat straw, and wheat bran. The UGCC yielded 10.67 mg/g FA, as quantified using the alkaline hydrolysis method, and was thus selected for downstream experiments (Fig. 2A). This value provides a reference for the theoretical maximum FA release from UGCC. Pretreatment is a critical step in the conversion of lignocellulose biomass into chemicals, by disrupting the recalcitrant structure of biomass and enhancing accessibility for downstream processes like enzymatic hydrolysis [33, 34]. In comparison to other methods, ultrafine grinding has emerged as a promising pretreatment technology for lignocellulose biomass due to its unique benefits, including particle densification, high surface area, enzymatic accessibility, energy efficiency, and environmental safety, offering a middle ground with both efficiency and environmental benefits [35–37].

The bifunctional xylanase/feruloyl esterase (rXyn10A/Fae1A) from the bacterial consortium EMSD5 was employed to construct the upstream xylan hydrolysis module, named the XF strain (Fig. 2B). Previous studies have shown that the recombinant rXyn10A/Fae1A exhibits high efficiency in converting various agricultural byproducts into FA and xylo-oligosaccharides simultaneously, even at a low enzyme dosage, achieving the highest yield reported for enzymatic conversion from xylan to FA (7.31 mg/g-SECC) through its inter-domain synergism [38]. Here, AlphaFold3-based ab initio prediction revealed that rXyn10A/Fae1A is composed of four domains: a xylanase domain from GH10, a carbohydrate esterase domain from CE1, and two carbohydrate-binding modules (CBMs) from families 13 and 2 (Fig. 2C). Notably, the spatial proximity of the GH10 and CE1 domains suggests potential inter-domain synergy, which could enhance the hydrolysis of complex lignocellulosic substrates [38].

**Fig. 3:**
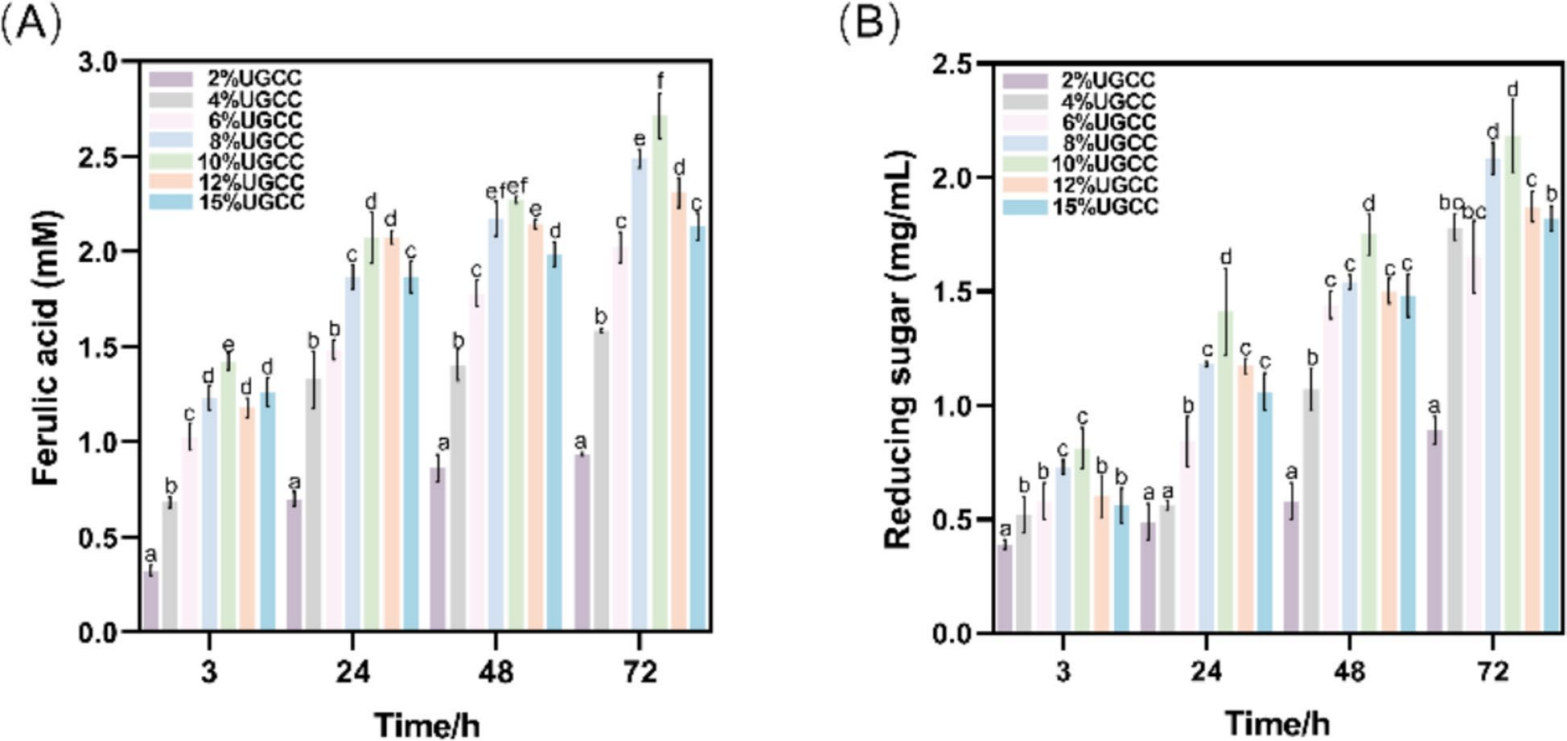
Optimization of natural substrate concentration for strain XF. **A** Concentration of FA. **B** Reducing sugar content

Subsequently, a 72-h fermentation experiment was conducted using 20 g/L UGCC as the substrate to evaluate the xylanolytic capacity of the recombinant strain XF. The results indicated that strain XF released 0.94 mM FA with a substrate yield of 9.12 mg/g, which is equivalent to releasing 85.50% of the alkali-hydrolyzable FA from UGCC. The total concentration of reducing sugars in the fermentation broth of strain XF was 0.84 mg/mL (Fig. 2D). TLC and HPAEC-PAD analyses identified xylobiose (0.29 mg/mL) as the major hydrolysis product, along with minor amounts of xylose (0.023 mg/mL) and other xylo-oligosaccharides (including xylotriose at 0.028 mg/mL) (Fig. 2E, F). The surface morphological changes of UGCC at different fermentation times were observed using SEM (Fig. 2H). In contrast to the unfermented UGCC, which exhibited a compact and relatively smooth surface with intact blocky structures, the XF-treated samples showed progressive structural disruption over time. After 24 h of fermentation, the surface became roughened with visible cracks and fissures. By 48 h, extensive fragmentation and delamination were observed, with the appearance of small, loose debris. After 72 h, the original compact architecture was largely replaced by irregular, porous fragments. These morphological modifications are consistent with the enzymatic hydrolysis of xylan and the release of FA, as previously reported in studies on lignocellulose degradation by xylanase and feruloyl esterase [39]. The gradual breakdown of the substrate surface likely enhances enzyme accessibility and facilitates further hydrolysis. To monitor the chemical structural changes of UGCC resulting from enzymatic hydrolysis, FTIR analysis was performed during fermentation (Fig. 2G). After 72 h of fermentation, the vibration peak at 1733 cm⁻^1^, which is attributed to C = O stretching vibrations of ester linkages in hemicellulose and lignin [40], was markedly diminished, indicating substantial cleavage of ester bonds. The vibration peak at 900 cm⁻^1^, associated with β-glycosidic linkages in cellulose and xylan, was also significantly weakened, suggesting depolymerization of the polysaccharide backbone. Meanwhile, the vibration peak at 1645 cm⁻^1^, corresponding to C = C stretching of aromatic rings or unsaturated structures, showed an increase in intensity, which may reflect the accumulation of lignin-derived aromatic compounds or the formation of new unsaturated bonds following structural disruption [41]. These spectral changes collectively indicate that the XF strain effectively degrades both carbohydrate and aromatic components of UGCC, consistent with the bifunctional activity of rXyn10A/Fae1A. To optimize FA production from UGCC, the substrate loading was varied from 20 to 150 g/L (2% to 15% w/v) and evaluated after 72 h of fermentation with strain XF. The FA concentration increased with substrate loading up to 100 g/L, reaching 2.71 mM, while the reducing sugar content peaked at 2.18 mg/mL (Fig. 3A, B). When the substrate loading was further increased to 150 g/L, no significant increase in FA or reducing sugar yield was observed, likely due to impaired mixing efficiency and mass transfer limitations in shake-flask cultivation. Therefore, 100 g/L UGCC (10% w/v) was selected as the optimal substrate loading for subsequent experiments, representing a balance between maximizing product titer and maintaining operational feasibility under shake-flask conditions. Although higher solid loadings are theoretically desirable for industrial applications, they present practical challenges in laboratory-scale cultivation, including reduced oxygen transfer, heterogeneous mixing, and sampling reliability [42]. Future scale-up studies may benefit from fed-batch strategies or bioreactor systems with improved mass transfer to overcome these limitations. Previous studies have demonstrated that the synergy between xylanase and ferulate esterase can efficiently facilitate xylan hydrolysis and release of FA [43–45]. Specifically, endo-β*-*1,4-xylanase catalyzes the cleavage of the xylan framework to generate ferulylated xylo-oligosaccharides (FXOS), while ferulate esterase tends to bind to FXOS [46–48]. This cooperative mechanism effectively disrupts xylan–lignin cross-linking, leading to FA yields that are several-fold (2.7 to 17) higher than those achieved using individual enzymes [49, 50]. In comparison with multi-enzyme systems, naturally evolved multifunctional enzymes have demonstrated incomparable advantages in practical production, including intrinsic domain synergy and simplified process optimization [51]. The bifunctional enzyme rXyn10A/Fae1A has demonstrated a remarkable capability to efficiently hydrolyze diverse agricultural byproducts to yield 71.90% of the total alkali-hydrolyzable FA from de-starched wheat bran [38]. Furthermore, a novel trifunctional endoxylanase/endoglucanase/feruloyl esterase Bi76 from *Bacteroides intestinalis* DSM 17393 exhibited a high capacity in producing FA, releasing 5.20 mg/g-substrate from corn bran with a corresponding conversion rate of 44.32% [52]. In this study, the xylan hydrolysis module XF, constructed by heterologously expressing rXyn10A/Fae1A in *K. phaffii*, released 85.50% of the alkali-hydrolyzable FA from UGCC, further demonstrating the versatility and efficiency of rXyn10A/Fae1A. In subsequent studies, we will analyze the protein structures of rXyn10A/Fae1A to elucidate its intramolecular synergistic mechanism and then modify the enzyme using structure-based and AI-driven approaches to boost its catalytic performance, aiming to further enhance the efficiency of rXyn10A/Fae1A and increase the FA yield of the xylan hydrolysis module.

### Construction of the downstream vanillin-producing module

**Fig. 4:**
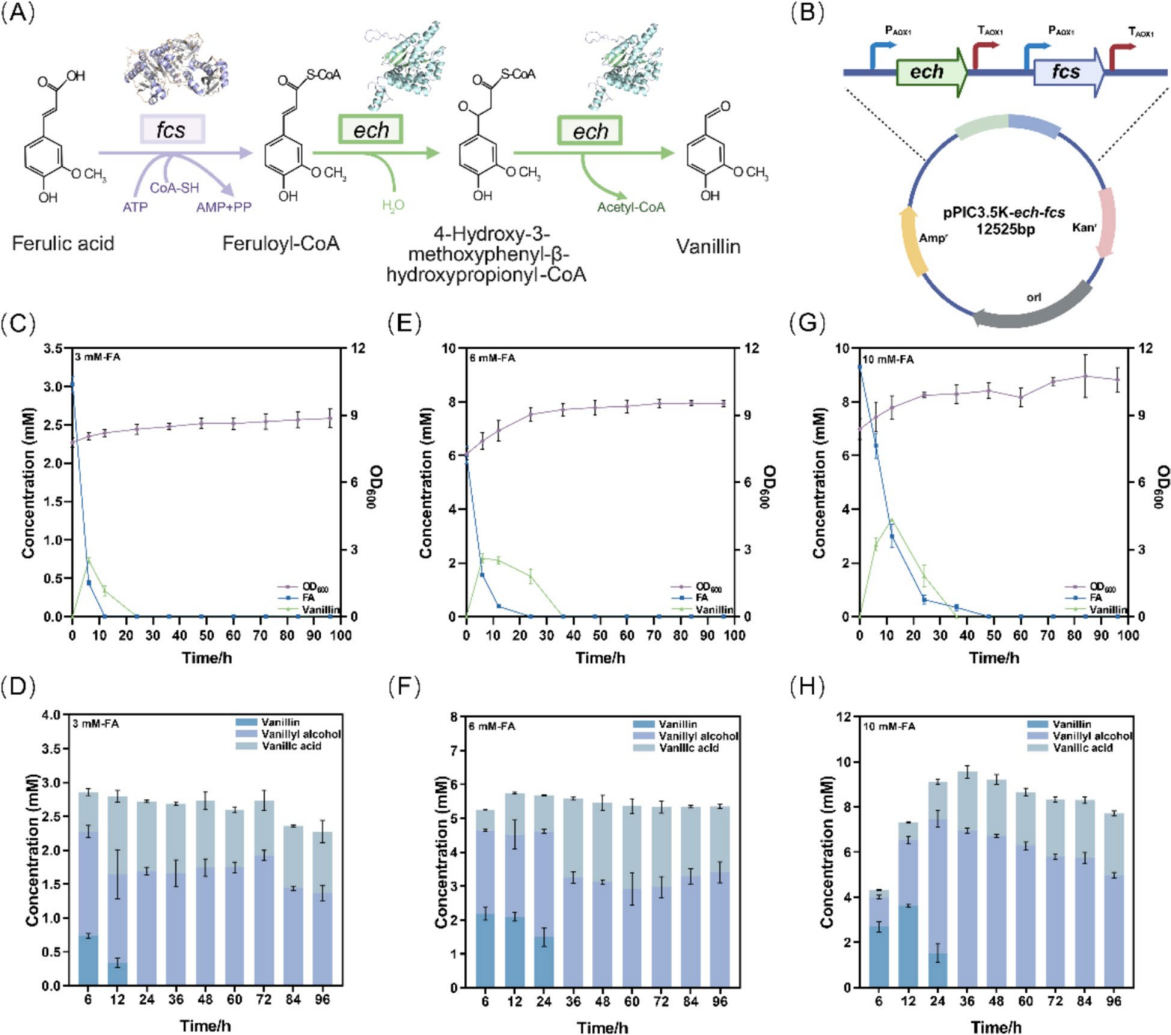
Construction of the downstream vanillin-producing module strain EF. **A** Synthetic pathway from FA to vanillin. **B** Schematic diagram of recombinant plasmid pPIC3.5 K-*ech*-*fcs*. **C** Vanillin production from 3 mM FA. **D** Concentration of vanillin and its derivatives at 3 mM FA. **E** Vanillin production from 6 mM FA. **F** Concentration of vanillin and its derivatives at 6 mM FA. **G** Vanillin production from 10 mM FA. **H** Concentration of vanillin and its derivatives at 10 mM FA

The downstream vanillin production module strain EF, constructed with *fcs* and *ech* genes from *Amycolatopsis* sp. ATCC 39116, utilizes FA as the substrate (Fig. 4A, B). Within the CoA-dependent bioconversion pathway, FCS and ECH serve as excellent biocatalysts for efficiently converting FA to vanillin [53]. The main microorganisms naturally possessing *fcs* and *ech* genes are *Pseudomonas*, *Amycolatopsis*, and *Streptomyces* species [54], among which *Amycolatopsis* sp. ATCC 39116 produced 19.30 g/L vanillin, exhibiting the highest molar conversion rate (94.90%) [55]. To evaluate the vanillin-producing capacity of the EF module, FA was supplied at three different initial concentrations: 3 mM, 6 mM, and 10 mM. The 10 mM concentration was selected based on preliminary tolerance tests as a safe upper limit for *K. phaffii* growth. The 6 mM concentration corresponds to the theoretical maximum FA release from UGCC determined by alkaline hydrolysis, and the 3 mM concentration reflects the actual FA yield from UGCC by the XF module under optimized conditions. As shown in Fig. 4C–H, the EF strain efficiently converted all three concentrations to vanillin and its derivatives (mainly vanillyl alcohol and vanillic acid), achieving molar conversion rates of 95.00% (3 mM, 6 h), 95.67% (6 mM, 12 h), and 95.60% (10 mM, 36 h), respectively. These results confirm that the EF module operates efficiently across the full range of FA concentrations relevant to the co-culture system. The slight accumulation of byproducts was observed in all conditions, consistent with the endogenous alcohol dehydrogenase (ADHs), aldehyde dehydrogenase (ALDRs), and aldo-keto reductase (AKRs) activities in *K. phaffii* [56].

Inhibiting the bioconversion of byproducts such as vanillic acid and vanillyl alcohol is an important strategy to further enhance the conversion and titers of the target compound vanillin in microbial cell factories [57]. Previous research indicated that rational targeted deletions of the six genes encoding AKRs and ADHs in *E. coli*, followed by the expression of a recombinant carboxylic acid reductase, could significantly enhance the accumulation of aromatic aldehydes [58, 59]. Xin et al. knocked out seven genes in *S. cerevisiae* BY4742, including those encoding AKRs, ALDRs, short-chain dehydrogenase/reductases (SDRs) and medium-chain dehydrogenase/reductases (MDRs), and further overexpressed vanillyl alcohol oxidase, resulting in the retention of 81.80% of vanillin within 48 h of fermentation [60]. Mo et al. simultaneously deleted more than ten oxidoreductase-encoding genes in *S. cerevisiae* BY4741, thereby enhancing its ability to accumulate vanillin [61]. We have conducted an exhaustive search of the *K. phaffii* genome and identified 20 genes encoding ADHs, AKRs, and ALDRs. In subsequent studies, we will knock out these genes sequentially to further promote the accumulation of the vanillin produced by strain EF. In addition, the complex interplay between various enzymes within the cellular environment also complicates efforts to specifically target and inhibit the degradation pathways of vanillin, and the regulatory mechanisms have not been fully understood. Despite progress in suppressing vanillin degradation, achieved by lowering reaction temperatures, shortening reaction times, and improving whole-cell catalytic efficiency, completely preventing it remains a significant challenge [62].

### Construction and optimization of the co-culture system for xylan hydrolysis and vanillin synthesis

**Fig. 5:**
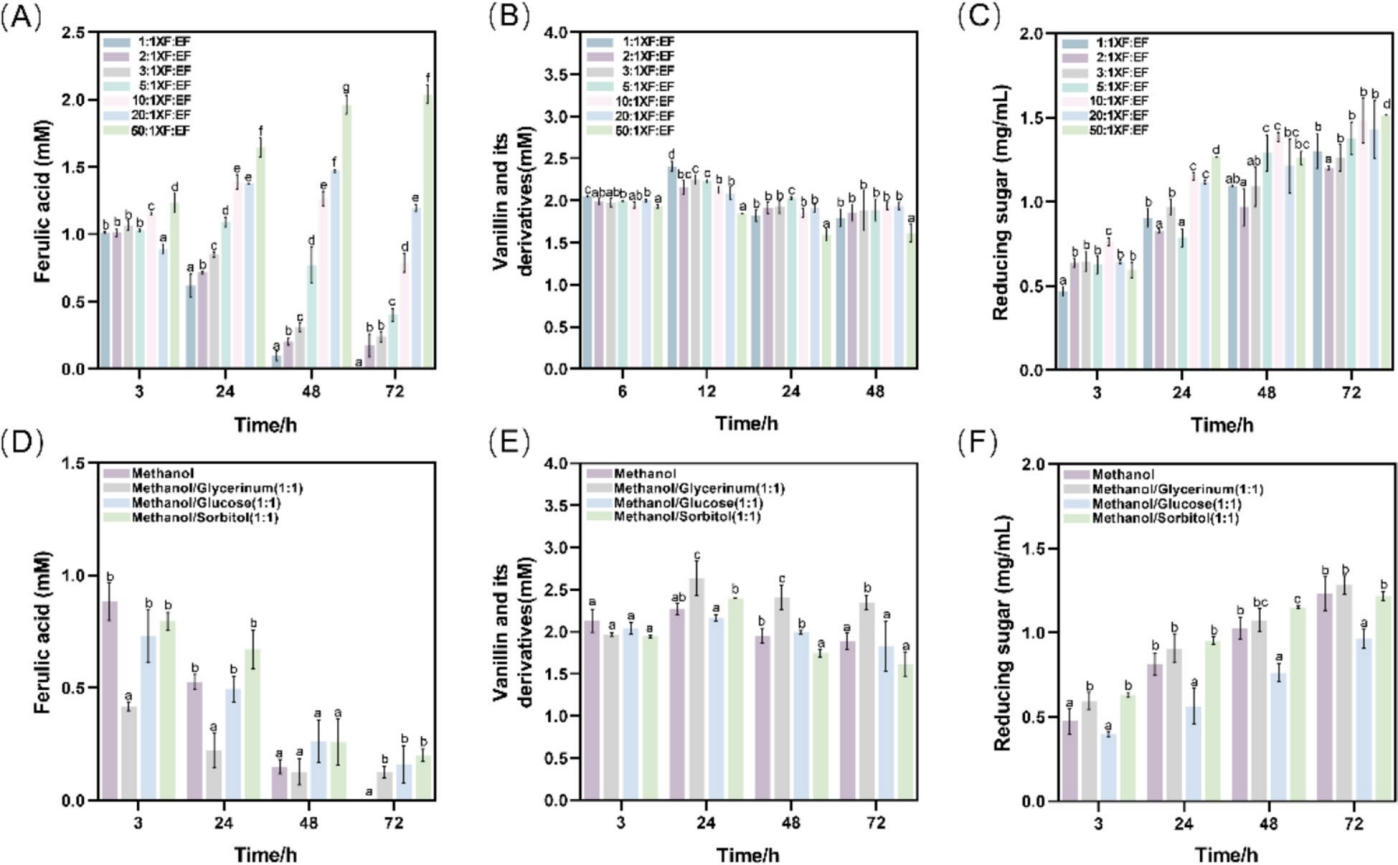
Optimization of inoculation ratios (XF: EF) and carbon sources in the co-culture system. **A** Concentration of FA under different inoculation ratios (XF: EF). **B** Concentration of vanillin and its derivatives under different inoculation ratios (XF: EF). **C** Reducing sugar content under different inoculation ratios in the co-culture system (XF: EF). **D** Concentration of FA under different carbon sources. **E** Concentration of vanillin and its derivatives under different carbon sources. **F** Reducing sugar content under different carbon sources

Modular co-culture systems have been widely recognized as an effective strategy to alleviate metabolic burden, balance biosynthetic flux, and minimize pathway crosstalk compared to single-strain systems [63–65]. Co-cultivation was employed to integrate upstream xylan hydrolysis (strain XF) with downstream vanillin synthesis (strain EF) for efficient biosynthesis. The feasibility of this modular co-culture system was first evaluated using 100 g/L UGCC as the substrate. Strains XF and EF were inoculated at different ratios (XF: EF = 1:1, 2:1, 3:1, 5:1, 10:1, 20:1, and 50:1) to determine the optimal population balance for vanillin production. Among these ratios, the 1:1 inoculation group produced the highest concentration of vanillin and its derivatives at 24 h, reaching 2.40 mM, with a molar conversion rate of 43.60%, and exhibited the reducing sugar content of 1.30 mg/mL at 72 h (Fig. 5B, C). Moreover, no intermediate product FA was detected after 48 h of fermentation, indicating that the FA released by the upstream strain XF was completely utilized by the downstream strain EF at the 1:1 inoculation ratio (Fig. 5A). This observation suggests an efficient metabolic “hand-off” between the two modules, where the rate of FA release by XF is well matched with the uptake and conversion capacity of EF, thereby minimizing intermediate accumulation and improving overall pathway efficiency. The titers of vanillin and its derivatives peaked within 24 h before declining, primarily due to the depletion of readily releasable FA from UGCC, which limited precursor availability. In addition, the multi-step, redox-dependent conversion of vanillyl alcohol to vanillic acid in *K. phaffii* is not strictly quantitative, likely contributing to the changes in product distribution during prolonged cultivation. To further enhance vanillin production, carbon sources in the co-culture system were optimized using three mixtures: methanol-glycerol, methanol-glucose, and methanol-sorbitol (1:1 ratio of methanol to other carbon sources). Among these, the methanol-glycerol group achieved the highest production of vanillin and its derivatives, reaching 2.63 mM at 24 h with a 1.09-fold increase in yield and a 47.90% molar conversion rate from UGCC (Fig. 5E). Concurrently, this group exhibited a reducing sugar content of 1.28 mg/mL at 72 h (Fig. 5F). Notably, the dynamic profile of FA during fermentation further supported this trend. In the methanol–glycerol group, FA was rapidly consumed and remained at a low level throughout the fermentation process, indicating an improved balance between upstream FA release and downstream conversion capacity (Fig. 5D).

A co-culture system for xylan hydrolysis and vanillin synthesis was successfully constructed in this study, enabling the high-value utilization of agricultural byproducts. However, it still faces several challenges. In addition to the issue of vanillin degradation, the efficiency, stability and sustainability of the co-culture system are also urgent problems that need to be addressed to unleash its full potential [63]. In artificial co-culture systems, different microbial modules establish mutually beneficial cooperative relationships by complementing metabolic pathways, engaging in symbiotic relationships, or alternating interactions [66, 67]. Adjusting the inoculation ratio and the carbon source ratio of the culture medium are simple strategies to optimize the cooperative mechanism, allowing the microbial community to function better, thereby improving the stability and efficiency of the entire system [68]. However, in this study, despite attempting different inoculation ratios and carbon sources to improve the conversion efficiency from xylan to vanillin, the results were suboptimal, and more precise and effective methods are needed to achieve the goals. Sequential inoculation represents a promising strategy to enhance FA availability prior to vanillin conversion, thereby avoiding premature FA consumption or metabolic competition. Additionally, enzyme engineering to improve the catalytic efficiency of rXyn10A/Fae1A could further boost FA release from lignocellulosic substrates. In subsequent research, the design of carbon source separation between modules will be applied in this system to reduce competition for carbon sources between the two microbial populations and enhance system stability. Metabolic flux optimization will also be conducted for each module within the co-culture system, including enhancing key enzyme activities, facilitating nutrient exchange, developing novel signal transduction mechanisms, and promoting biocompatibility, ultimately aiming to achieve higher vanillin yields [63]. Furthermore, a deeper understanding of microbial interactions between the upstream xylan-hydrolysis module XF and downstream vanillin-producing module EF in the co-culture system will be achieved by integrating transcriptomics and proteomics analyses, including microbial community structure, functional gene expression regulation, material exchange and energy transfer, and quorum sensing signals. This understanding will enable the design of more precise and intelligent methods to enhance the synergy between these modules, thereby improving the efficiency of xylan conversion to vanillin in the co-culture system.

## Conclusion

A novel dual-module co-culture system, integrating an upstream xylan-hydrolysis module and a downstream vanillin-producing module, was successfully designed and constructed to convert agricultural byproduct-derived xylans into food-grade vanillin. Through the optimization of initial inoculation ratios and carbon-source composition, the co-culture system achieved 2.63 mM vanillin and its derivatives (vanillyl alcohol and vanillic acid) from UGCC with a 47.90% molar conversion rate. This study establishes a strategy for agricultural byproducts valorization through resource recycling and value-added conversion, offering a promising framework for industrial biosynthesis of food-grade vanillin.

## Data Availability

Data will be made available on request.

## Abbreviations

FA: Ferulic acid
ρ-CA: ρ-Coumaric acid
CA: Caffeic acid
XOS: Xylo-oligosaccharides
FAE: Feruloyl esterase
FCS: Feruloyl-CoA synthetase
ECH: Enoyl-CoA hydratase
GRAS: Generally Recognized As Safe
BMGY: Buffered glycerol-complex medium
BMMY: Buffered methanol-complex medium
YNB: Yeast nitrogen base
LLB: Low-Luria–Bertani
UGCC: Ultrafine-grinding corncob
Corncob: Corncob powder
SECC: Steam-exploded corncob
UGCS: Ultrafine-grinding corn stover
SECS: Steam-exploded corn stover
UGRS: Ultrafine-grinding rice straw
DSWB: De-starched wheat bran
UGWS: Ultrafine-grinding wheat straw
rXyn10A/Fae1A: Xylanase/feruloyl esterase
FXOS: Ferulylated xylo-oligosaccharides
ADHs: Alcohol dehydrogenase
ALDRs: Aldehyde dehydrogenase
AKRs: Aldo-keto reductase
SDRs: Short-chain dehydrogenase/reductases
MDRs: Medium-chain dehydrogenase/reductases
